# A review of sugar consumption from nationally representative dietary surveys across the world

**DOI:** 10.1111/jhn.12338

**Published:** 2015-10-10

**Authors:** K. J. Newens, J. Walton

**Affiliations:** ^1^ Sugar Nutrition UKSomerset House, StrandLondonUK; ^2^ School of Food and Nutritional SciencesUniversity College CorkCorkIreland

**Keywords:** added sugars, dietary surveys, sucrose, sugar consumption, total sugars

## Abstract

**Background:**

Government and health organisations worldwide have recently reviewed the evidence on the role of dietary sugars in relation to health outcomes. Hence, it is timely to review current intakes of dietary sugars with respect to this guidance and as a benchmark for future surveillance.

**Methods:**

This review collates data from nationally representative dietary surveys across the world and reports estimates of intakes of total and added sugars, and sucrose in different population subgroups. Total sugars includes all mono‐ and disaccharides; namely, glucose, fructose, lactose, sucrose and maltose. Added and free sugars differ in the quantity of natural sugars included in their definitions. Free sugars include sugars naturally present in honey, syrups, fruit juices and fruit juice concentrates, whereas added sugars typically only refer to those added during processing.

**Results:**

Most countries reported intakes of total sugars, with fewer reporting intakes of added sugars and sucrose. No country reported intakes of free sugars. The available data suggest that total sugars as a percentage of energy were highest in the infant (<4 years), with mean values ranging from 20.0% to 38.4%, and decreased over the lifespan to 13.5–24.6% in adults. Intakes of added sugars were higher in school‐aged children and adolescents (up to 19% of total energy) compared to younger children or adults.

**Conclusions:**

Further research into the dietary patterns contributing to added sugars intake in children and adolescents is warranted. It would also be beneficial to policy guidance if future dietary surveys employed a uniform way of expressing sugars that is feasible to measure and has public health significance.

## Introduction

The role of dietary sugars has become an increasingly active public health issue over recent years. This has prompted many government and health organisations worldwide to review the evidence on the role of dietary sugars in relation to obesity and its related disorders and also to dental health. In 2010, The European Food Safety Authority published its scientific opinion on dietary reference values for carbohydrates and dietary fibre and was unable to set an upper level for sugars intake as a result of insufficient evidence in relation to body weight, cardiovascular risk factors, type 2 diabetes, nutrient density of the diet or dental caries 1 . The report did find evidence between an increased risk of dental caries and the *frequency* of consumption of sugar‐containing foods but not with the *quantity* of sugars consumed. Additionally, there was some evidence that high intake of *sugar‐sweetened beverages* might contribute to weight gain but data were inconsistent in relation to solid foods again making it difficult to set a quantitative upper level for intake of sugars 1. These findings are consistent with previous guidance from the US Institute of Medicine 2 that were unable to set an upper limit for sugars intake but suggested a maximal intake for added sugars (defined as those sugars incorporated into foods and beverages during production) of 25% of energy for the purpose of avoiding the low nutrient intakes associated with an intake of added sugars above this level.

Earlier this year, the World Health Organization (WHO) published its updated guideline on free sugars intake for adults and children in relation to body weight and oral health; free sugars being defined as monosaccharaides and disaccharides added to foods and beverages by the manufacturer, cook or consumer and sugars naturally present in honey, syrups, fruit juices and fruit juice concentrates. The recommendations were: (i) a reduced intake of free sugars throughout the life‐course (strong recommendation); (ii) the reduction of intake of free sugars to <10% of total energy (TE) intake in both adults and children (strong recommendation); and (iii) a further reduction of free sugars to below 5% of total energy intake (conditional recommendation) 3. The report acknowledged that the excess body weight associated with free sugars intake results from excess energy intake and that the quantitative recommendations (<5%TE and <10% TE) are based on the relationship with dental caries only. Furthermore, it was acknowledged that the recommendation to limit free sugars intake to <5%TE is based on very low quality evidence from ecological studies.

The Scientific Advisory Committee of Nutrition in the UK has also reviewed the evidence between sugars, individual sugars, sugar‐sweetened foods and beverages, and sugar alcohol intake with respect to cardio‐metabolic, colorectal and oral health outcomes and findings and recommendations are expected to be issued later this year 4. Concurrently, the *Scientific Report of the 2015 Dietary Guidelines Advisory Committee (DGAC)* ^*(*^ 5 ^)^ has recently been issued for public consultation, which will provide the US government with a basis for developing national nutrition policy.

With revised dietary guidance becoming available regarding consumption of dietary sugars, it is timely to review current estimates in different countries in order to assess intakes against recommendations and to provide a benchmark for ongoing surveillance. Sugars can be estimated indirectly using supply or availability data (e.g. food balance sheets) or food purchase surveys, or more directly (individual assessments) using either prospective measurements such as food records, or retrospective methods such as food frequency questionnaires or recalls. Supply data for sugars have been reliably collated by the Food and Agricultural Organization (FAO) since 1961 6. Although these data provide a useful estimate of per capita availability of sugars, they are unable to account for wastage, which is known to be significant particularly in developed countries. Recent data from the UK demonstrate that approximately 15 million tonnes of food is thrown away in the UK each year 7 and US data suggest that food wastage accounted for almost 40% of the available US food supply in recent years 8. Furthermore, supply data are not suitable for assessing differences in consumption patterns for different subgroups of the population, such as age, sex or socio‐economic group 9, which is a key requirement for determining nutrition policy and assessing how different sectors of the population are achieving any devised targets. Direct dietary assessments via national food consumption surveys are carried out in many countries worldwide 10, 11, relying on tables of food composition data to estimate nutrient intakes from the foods recorded.

The number of terms used to describe sugars has resulted in difficulties with respect to providing comparisons between countries and also impacts on the ability to compare intakes with recommendations, risk factors or disease endpoints, and with the results of intervention studies. The term ‘total sugars’ includes all mono‐ and disaccharides; namely glucose, fructose, lactose, sucrose and maltose. Added and free sugars differ in the quantity of natural sugars included in their definitions. Free sugars include sugars naturally present in honey, syrups, fruit juices and fruit juice concentrates, whereas added sugars only refers to those added during processing. In the UK, the term ‘non‐milk extrinsic sugars’ (NMES) has been used, which is designed to be a measure of sugars that are not contained within the cell wall, excluding those in milk 4. The difference between NMES and free sugars is that NMES includes 50% of the fruit sugars from stewed, dried or canned fruit 12, whereas free sugars includes none. The term ‘sugar’ is also widely used and typically refers to table sugar (sucrose) but is also used in some instances to describe either total sugars or added sugars, which can lead to confusion for researchers, practitioners and thus the consumer.

The present study aims to review and collate the available data on sugars consumption across the world using the most recent nationally representative dietary surveys for each country, and to review these data with respect to the different population groups surveyed.

## Materials and methods

### Selection of data

Recent reviews of dietary surveys in both the developed world 10 and the developing world 11 were used to identify a list of countries that have undertaken dietary surveys and the dates that these surveys were performed. Nutrient and food intakes derived from national food consumption survey data are typically published in reports, rather than peer‐reviewed papers; hence, the data presented in the present review were primarily collected from various reports and publications of national dietary surveys. Accordingly, a formal systematic review was not performed and surveys were identified through searches of PubMed and Internet search engines using terms such as ‘national dietary survey’ and ‘sugar(s) consumption’. Surveys and reports known to the authors of the present study were also included. Studies were included if the data were (i) nationally representative; (ii) available in the public domain; (iii) summarised (no raw data were analysed); (iv) collected at the individual level; and (v) sugars intake was reported as a nutrient rather than a food‐group. There was no limit to the study size and data from all direct dietary assessment methodology were included (e.g. food diaries, diet recalls or food‐frequency questionnaires).

Authors of publications reporting findings from national dietary surveys in Korea, Brazil and Japan kindly replied to requests for assistance; however, in all cases (further) data on total or added sugar consumption had not been collected and were therefore not available to be included in the present review.

### Presentation of data

Values are reported for estimated intakes of energy, total sugars, added sugars, NMES or sucrose as available. Where sugars intake as a percentage of energy has not been provided, this was calculated using 0.016 or 0.017 MJ g^−1^). (depending on which is used in the country in question). Data are presented as provided in the reports and publications and, where possible, data have been divided by age range and not subdivided by sex. No statistical analysis has been performed on these datasets, the results are purely descriptive.

Graphs are presented to show the ranges of energy and sugars consumption for each age group. Each graph only contains one data point per country; means were calculated for this purpose. Because the graphs are derived from the available data, the number and range of countries represented will vary.

### Under‐reporting

Under‐reporting of food consumption can influence the accuracy of dietary intake data resulting in an underestimate of energy and nutrients. A small number of reports excluded under‐reporters from their studies (based on under‐reporting for total energy intake). Where these data are available, the percentage of under‐reporters in each country is indicated within the corresponding table (Tables 1, 2, 3, 4, 5). Other studies acknowledged the percentage of low energy reporters but did not exclude these subjects from their analyses.

**Table 1:** Energy and sugar intake of infants and young children (<4 years)

	Year	Age range (y)	*n*	Method	Energy		Total sugars				Added sugars^b^				Sucrose			
					MJ	(SD)	g	(SD)	%E*	(SD)	g	(SD)	%E*	(SD)	g	(SD)	%E*	(SD)
Australia	2011–12 36	2–3		24‐h recall	5.9		91.6		24.5									
	1995 16	2 (M)	93	24‐h recall							30.4		9.1					
		3 (M)	114	24‐h recall							27.0		7.6					
		2 (F)	114	24‐h recall							26.9		6.6					
		3 (F)	86	24‐h recall							28.9		10.6					
Canada	2004 37	1–3	2117	24‐h recall	6.2	(4.0)	100	(92)	27.1									
Finland	2003–5 38	1BF (M)	55	3 day record	2.8	(0.8)	43	(16)	25.7						10.5	(5.9)	6.3	
		1FF (M)	257	3 day record	3.9	(0.7)	69	(18)	30.0						13.0	(9.4)	5.6	
		2 (M)	112	3 day record	4.6	(1.0)	78	(23)	29.0						34.3	(16.9)	12.5	
		3 (M)	236	3 day record	5.4	(1.0)	90	(27)	29.0						41.7	(18.3)	13.2	
		1BF (F)	57	3 day record	2.6	(0.8)	38	(15)	24.6						9.0	(6.0)	5.8	
		1FF (F)	198	3 day record	3.6	(0.6)	64	(16)	30.0						11.7	(8.0)	5.4	
		2 (F)	118	3 day record	4.5	(0.9)	77	(21)	29.0						31.5	(14.7)	11.9	
		3 (F)	235	3 day record	5.0	(1.0)	85	(23)	29.0						40.3	(17.0)	13.5	
Iceland	2005–7 39	9 months	196	3 day record	3.1	(0.8)	43.9	(18)	23.4		3.5	(4.7)	1.9					
		1	170	3 day record	3.5	(0.8)	41.5	(17)	20.0		8.7	(6.4)	4.2					
		3	225	3 day record	5.8	(1.1)					26	(13)	7.5					
Ireland	2010–2011 40	1	126	4 day record	4.2	(0.9)	69.6	(22)	26.0									
		2	124	4 day record	4.7	(1.2)	74.3	(23)	27.9									
		3	126	4 day record	4.8	(0.9)	76.0	(20)	24.8									
Italy	2005–6 41	0–3	52	3 day record	4.7	(1.8)	71.4	(36)	24.9	8.8								
Netherlands^a^	2007–10 42	2–3 (M)	327	2 day record	5.7		124		36.1									
		2–3 (F)	313	2 day record	5.5		119		36.4									
Norway	2006–7 43	7 months–1	881	24‐h recall	5.1	(1.6)					12.2	(14.0)	4.0	4.1				
		14 months–2	1674	24‐h recall	5.7	(1.5)					22.5	(16.0)	6.7	4.2				
UK^b^	2008–12 (Public Health England 2013)	1.5–3	604	4 day record	4.8	(1.1)	75.6	(25)	25.1	5.9	36.1	(19.3)	11.9	5.4				
	2011 14	4–6 months	295	4 day record	2.9	(0.7)	70.9	(17)	38.4	4.9	8.1	(6.7)	4.3	3.3				
		7–9 months	698	4 day record	3.3	(0.7)	67.8	(16)	32.7	6.0	12.8	(9.3)	6.2	4.8				
		10–11 months	429	4 day record	3.6	(0.8)	66.7	(18)	29.4	5.7	14.3	(9.5)	6.2	4.0				
		12–18 months	1261	4 day record	4.1	(0.9)	66.0	(19)	25.8	5.6	19.8	(12.1)	7.7	4.5				
US	2009–10 17	2–5	823	24‐h recall	6.4	(3.0)					51.6	(34.0)	13.4	8.6				

M, males only; F, females only; BF, breast‐milk fed; FF, formula fed. Data are represented as the mean, unless otherwise indicated.

Where percentage energy was not given, this was calculated (using 0.016/0.017 MJg^−1^.

a Median values are given for the Netherlands^42^.

b Non‐milk extrinsic sugars (UK only) have been designated as added sugars^14^.

**Table 2:** Energy and sugar intake of children (4–10 years)

	Assessment year	Age range (y)	*n*	Method	Energy		Total sugars (g)				Added sugars (g)				Sucrose (g)			
					MJ	(SD)	g	(SD)	%E	(SD)	g	(SD)	%E	(SD)	g	(SD)	%E	(SD)
Australia	2011–12 36	4–8		24‐h recall	7.1		103		23.5									
Austria^a^	2010–12 44	7–9 (M)	67	3 day record	8.0												12.0	
		10–12 (M)	83	3 day record	8.1												10.0	
		7–9 (F)	57	3 day record	8.0												13.0	
		10–12 (F)	81	3 day record	7.2												14.0	
Canada	2004 37	4–8	3235	24‐h recall	7.9	(4.5)	122	(114)	25.8									
Denmark	2003–6 45	4–14	669	7 day diary	8.3						58		12.0					
Finland	2008 38	4 (M)	307	3 day record	5.8	(1.1)	97	(26)	28.0						46.9	(18)	13.7	
		4 (F)	247	3 day record	5.5	(1.0)	92	(22)	29.0						43.6	(16)	13.6	
		6 (M)	364	3 day record	6.7	(1.2)	108	(28)	28.0						51.5	(19.9)	13.0	
		6 (F)	349	3 day record	6	(1.1)	99	(27)	28.0						48.7	(19.3)	13.8	
France	2005–7 46	3–17	1444	7 day record	7.4	(2.1)	98.6	(34)	22.2									
Germany	2006 47	6 (M)	106	3 day record	7.2	(1.4)	121.3	(40)	28.4									
		7–9 (M)	321	3 day record	7.8	(1.6)	126.5	(41)	27.1									
		10–11 (M)	199	3 day record	8.0	(1.8)	123	(42)	25.8									
		12 (M)	114	FFQ	10.6	(3.2)	167	(71.8)	26.4									
		6 (F)	102	3 day record	6.3	(1.3)	101	(29)	26.7									
		7–9 (F)	308	3 day record	7.0	(1.4)	114.7	(41)	27.6									
		10–11 (F)	198	3 day record	7.6	(1.6)	119.6	(47)	26.5									
		12 (F)	103	FFQ	9.3	(3.2)	154.5	(86)	27.8									
Iceland	2007 48	5	231	3 day record	6.3	(1.2)					34	(16)	9.0					
	2011–12 49	6	162	3 day record	6.5	(1.4)							11.2					
	2003–4 50	9	175	24‐h recall	8.2	(1.8)	125	(42)	25.4		66	(37)	13.4					
Ireland	2010–2011 40	4	124	4 day record	5.3	(1.0)	83.6	(25.1)	24.7									
	2003–2004 51	5–12	594		7.0	(1.5)	106.6	(34)	23.9	(5.3)	65.2	(30)	14.6	(5.4)				
Italy	2005–6 41	3–10	193	3 day record	8.0	(2.0)	86	(30)	17.0	(5.0)								
Netherlands	2007–10 52, 53	7–8 (M)	153	24‐h recall	8.1		141		29.2		87		18		84		17	(3)
		7–8 (F)	151	24‐h recall	8.4		140		27.8		83		18		78		16	(3)
	2005–6 42	4–6 (M)	327	2 day record	6.7		135		33.7									
		4–6 (F)	312	2 day record	6.2		129		34.8									
NZ	2002 54	5–6 (M)	383	24‐h recall	7.6		110		24.2						58.5		12.9	
		7–10 (M)	738	24‐h recall	9		130		24.2						70.8		13.2	
		5–6 (F)	309	24‐h recall	6.8		105		25.8						55.6		13.7	
		7–10 (F)	687	24‐h recall	7.8		115		24.5						63.6		13.6	
Norway	2000 55	4 (M)	206	24‐h recall	6.3	(1.5)					54.6	(22)	14.8	5.0				
		8–10 (M)	404	24‐h recall	8.6	(2.0)					80.7	(35)	16.0	5.8				
		4 (F)	185	24‐h recall	6.1	(1.2)					55.3	(24)	15.6	6.0				
		8–10 (F)	411	24‐h recall	7.7	(2.0)					78.8	(34)	17.5	6.3				
Sweden	2003 28	8–9	889	4 day diary	7.6	(1.7)	116		25.6						57	(25)	12.6	(4.2)
UK	2008–12 (Public Health England 2014)	4–10	1277	4 day diary	6.5	(1.4)	97.4	(31.8)	23.8	(5.6)	60.8	(27.4)	14.7	5.3				
USA	2009–10 17	6–11 (M)	588	24‐h recall	8	(3.3)	126	(1.9)	24.6									
		6–11 (F)	566	24‐h recall	7.6	(2.4)	120	(2.5)	24.8									
	2007–08 56	6–11	1107	24‐h recall	8	(4.3)					83.6	(83)	17	(13)				

M, males only; F, females only. FFQ, food frequency questionnaire. Data are represented as the mean, unless otherwise indicated.

Where percentage energy was not given, this was calculated (using 0.016/0.017 MJg^−1^ depending on country).

†Median values are given for the Netherlands.

‡Non‐milk extrinsic sugars (UK only) have been designated as added sugars.

a Data exclude under‐reporters (10.3%).

**Table 3:** Energy and sugar intake of adolescents (~12–18 years)

	Assessment year	Age range (y)	*n*	Method	Energy		Total sugars (g)				Added sugars (g)				Sucrose (g)			
					MJ	(SD)	g	(SD)	%E	(SD)	g	(SD)	%E	(SD)	g	(SD)	%E	(SD)
Australia	2011–12 36	9–13		24‐h recall	8.6		120.4		22.6									
		14–16		24‐h recall	9.2		121.7		21.4									
Austria	2010–12 44	13–14 (M)	19	3 day record	8.6												10	
		13–14 (F)	25	3 day record	7.5												14	
Belgium	2004 57	15–18	873	24‐h recall	9.4	(2.8)			24.2	(5.7)								
Canada	2004 37	9–13 (M)	2080	24‐h recall	10.3	(7.3)	155	(137)	25.1									
		14–18 (M)	2288	24‐h recall	12.1	(9.6)	173	(191)	24.1									
		9–13 (F)	1980	24‐h recall	8.5	(5.8)	129	(89)	25.6									
		14–18 (F)	2256	24‐h recall	8.6	(5.4)	126	(95)	24.6									
Germany	2006 47	13–14 (M)	214	FFQ	11.7	(3.8)	187.6	(83.1)	26.8									
		15–17 (M)	294	FFQ	14.3	(5.4)	221.3	(113)	25.9									
		13–14 (F)	230	FFQ	9.5	(2.7)	160.3	(70.9)	28.2									
		15–17 (F)	317	FFQ	9.9	(3.8)	175.1	(96.2)	29.6									
Iceland	2003–4 50	15	150	24‐h recall	10.3	(2.7)	167	(68)	27.2		101	(64)	16.5					
Ireland	2003–4 51	13–17	441	4 day record	8.3	(2.4)	108.5	(43)	20.4	(5)	65.7	(32)	12.4	(4.9)				
Italy	2005–6 41	10–18 (M)	108	3 day record	10.8	(3.1)	107.6	(53.7)	15.4	(4.7)								
		10–18 (F)	139	3 day record	8.7	(2.2)	88.4	(35.6)	15.8	(5.2)								
Netherlands	2007–10 52, 53	9–13 (M)	351	24‐h recall	9.8		156		26.8		97		17		85		16	(3)
		14–19 (M)	352	24‐h recall	11		157		24.0		100		16		85		14	(3)
		9–13 (F)	352	24‐h recall	8.4		134		26.7		90		18		82		16	(3)
		14–19 (F)	354	24‐h recall	8.4		126		25.1		75		15		70		13	(3)
NZ	2008–9 58	15–18 (M)	326	24‐h recall	11.2		143		21.4						71.8		10.7	
		15–18 (F)	373	24‐h recall	7.9		118		25.2						62.7		13.4	
	2002 54	11–14 (M)	576	24‐h recall	10.5		144		22.9						76.6		12.2	
		11–14 (F)	582	24‐h recall	8.4		124		24.7						65.2		13.0	
Norway	2000 55	12–14 (M)	492	24‐h recall	9.5	(3.5)					101.6	(58)	18.2	(7.6)				
		12–14 (F)	517	24‐h recall	8	(2.6)					88	(42)	18.6	(6.7)				
Sweden	2003 28	11–12	1016	4 day diary	7.4	(2.1)	106		24.1						55	(30)	12.3	(5)
UK	2008–12 (Public Health England 2014)	11–18	1497	4 day diary	7.48	(2.17)	103.4	(44.8)	21.7	(6.6)	74.2	(39.8)	15.4	(6.4)				
US	2009–10 17	12–19 (M)	672	24‐h recall	10.6	(7.9)	161	(132)	23.8									
		12–19 (F)	593	24‐h recall	7.6	(4.5)	117	(107)	24.1									
	2007–8 56	12–17	869	24‐h recall							89.9	(103)	17.3	(20.6)				

M, males only; F, females only. FFQ, food frequency questionnaire. Data are represented as the mean, unless otherwise indicated.

Where % energy was not given, this was calculated (using 0.016/0.017 MJg^−1^).

†Median values are given for the Netherlands.

‡Non‐milk extrinsic sugars (UK only) have been designated as added sugars.

**Table 4:** Energy and sugar intake of adults

	Assessment year	Age range (y)	*n*	Method	Energy		Total sugars				Added sugars				Sucrose			
					MJ	(SD)	g	(SD)	%E	(SD)	g	(SD)	%E	(SD)	g	(SD)	%E	(SD)
Australia	2011–12 36	19–30		24‐h recall	9.5		115.3		19.8									
		31–50		24‐h recall	8.87		105.2		19.3									
		51–70		24‐h recall	8.29		93.9		18.2									
Austria^a^	2010–12 44	18–24 (M)	17	24‐h recall	10.1												10	
		25–50 (M)	87	24‐h recall	9.1												10	
		51–64 (M)	44	24‐h recall	9.4												8	
		18–24 (F)	37	24‐h recall	10.1												12	
		25–50 (F)	143	24‐h recall	9.1												11	
		51–64 (F)	52	24‐h recall	9.4												10	
Belgium	2004 57	19–59	873	24‐h recall	8.9	(3.1)			20.5	(5.9)								
Brazil	2008–9 59	10+	34003	2 day diary	8.0								7.2					
Canada	2004 37	19–30 (M)	1804	24‐h recall	11.5	(8.4)	137	(127)	20.0									
		31–50 (M)	2596	24‐h recall	10.5	(9)	117	(153)	18.6									
		51–70 (M)	2550	24‐h recall	9.2	(6.6)	102	(101)	18.5									
		19–30 (F)	1854	24‐h recall	8	(6.1)	107	(129)	22.5									
		31–50 (F)	2686	24‐h recall	7.7	(6.5)	92	(104)	19.9									
		51–70 (F)	3200	24‐h recall	7.1	(5.2)	85	(113)	20.0									
Denmark	2003–6 45	15–75	2578	7 day diary	9.1						48		9.0					
France^b^	2005–7 46	18–79	1918	7 day record	9.1	(2.5)	95	(40)	17.6									
Finland	2007 60	25–64 (M)	730	48 h recall	9.2	(3.0)									53	(37)	9.7	(5.9)
		25–64 (F)	846	48 h recall	6.8	(2.0)									43	(26)	10.5	(5.1)
Germany	2005–6 61	14–80 (M)	7093	24‐h recall	10.9		124		19.3									
		14–80 (F)	8278	24‐h recall	6.8		113		23.6									
Iceland	2010–11 62	18–30 (M)	131	24‐h recall	11	(3.7)	129	(54)	19.6		76	(47)	12					
		31–60 (M)	350	24‐h recall	10	(3.1)	105	(57)	17.5		56	(47)	9.1					
		18–30 (F)	119	24‐h recall	7.9	(2.1)	108	(47)	22.8		55	(41)	11.3					
		31–60 (F)	394	24‐h recall	7.5	(2.2)	86	(42)	119.2		40	(32)	8.5					
Ireland	2008–10 63	18–64	1274	4 day record	8.6	(2.8)	91.6	(44)	16.6	(5.7)								
	1997–99 51	18–64	1097	7 day record	10.2	(2.8)	108.3	(45)	16.8	(4.8)	61.9	(38)	9.4	(4.3)				
Italy	2005–6 41	18–65 (M)	1068	3 day record	10	(2.7)	86	(38)	13.5	(4.7)								
		18–65 (F)	1245	3 day record	8.1	(2.2)	79.5	(33)	15.4	(5.1)								
Netherlands	2007–10 52, 53	19–30 (M)	356	24‐h recall	11.5		145		21.1									
		31–50 (M)	348	24‐h recall	11.1		122		18.4									
		19–50 (M)	703	24‐h recall							74		12		67		11	(6)
		51–69 (M)	351	24‐h recall	10		103		17.2		53		10		52		9	(5)
		19–30 (F)	347	24‐h recall	8.4		116		23.2									
		31–50 (F)	351	24‐h recall	8.2		102		20.9									
		19–50 (F)	698	24‐h recall							57		12		55		11	(5)
		51–69 (F)	353	24‐h recall	7.8		90		19.5		42		9		45		10	(5)
NZ	2008–9 58	19–30 (M)	284	24‐h recall	11.9		147		20.6						73.2		9.6	
		31–50 (M	598	24‐h recall	11.5		133		19.4						64.5		8.8	
		51–70 (M)	378	24‐h recall	9.4		108		19.3						49		8.2	
		19–30 (F)	434	24‐h recall	8.4		123		24.4						65.8		12.3	
		31–50 (F)	746	24‐h recall	7.9		98		20.7						45.3		9	
		51–70 (F)	517	24‐h recall	7.2		95		22.1						40.4		8.8	
Norway	2010–11 64	18–70	1787	24‐h recall	9.4	(3.3)					42	(38)	7.3	(5.4)				
Sweden	2010–11 65	18–80	1797	4 day diary	8.3	(2.6)	87.8		17.9		48	(30)	9.6	(4.6)	38.7	(24.7)	7.7	(3.7)
UK	2008–12 (Public Health England 2014)	19–64	2697	4 day diary	7.8	(2.5)	95.1	(45.3)	19.1	(6.5)	58.8	(39.7)	11.5	(6.1)				
US	2009–10 17	20–29 (M)	450	24‐h recall	11	(7.1)	146	(132)	22.2									
		30–39 (M)	455	24‐h recall	11.5	(4)	143	(143)	20.9									
		40–49 (M)	481	24‐h recall	11.4	(6.7)	141	(94)	20.7									
		50–59 (M)	470	24‐h recall	10.4	(5)	122	(152)	19.7									
		60–69 (M)	449	24‐h recall	9.2	(3.5)	108	(87)	19.6									
		20–29 (F)	524	24‐h recall	8.2	(5.2)	120	(105)	24.6									
		30–39 (F)	499	24‐h recall	7.7	(2.9)	104	(71)	22.7									
		40–49 (F)	555	24‐h recall	7.5	(5.8)	105	(118)	23.4									
		50–59 (F)	429	24‐h recall	7.4	(3.3)	100	(70)	22.7									
		60–69 (F)	453	24‐h recall	7.2	(3.2)	96	(70)	22.4									
	2007–08 56	18–34	1518	24‐h recall							92.3	(160)	16.3	(23)				
		35–54	1832	24‐h recall							80.7	(150)	14.3	(26)				
		55+	2286	24‐h recall							54.8	(67)	11.8	(9.6)				

M, males only; F, females only. Data are represented as the mean, unless otherwise indicated.

Where % energy was not given, this was calculated (using 0.016/0.017 MJg^−1^).

†Median values are given for the Netherlands.

‡Non‐milk extrinsic sugars (UK only) have been designated as added sugars.

a Data exclude under‐reporters (9.1%).

b Data exclude under‐reporters (no values given).

**Table 5:** Energy and sugar intake of older adults (>60 years)

Country	Assessment year	Age range (y)	*n*	Method	Energy		Total sugars (g)				Added sugars (g)				Sucrose (g)			
					MJ	(SD)	g	(SD)	%E	(SD)	G	(SD)	%E	(SD)	g	(SD)	%E	(SD)
Australia	2011–12 36	71+		24‐h recall	7.29		92.2		20.1									
Austria^a^	2010–12 44	65–80 (M)	76	24‐h recall	8												8	
		65–80 (F)	100	24‐h recall	7												9	
Belgium	2004 57	60–74	822	24‐h recall	7.8	(2.2)			19.2	(5.7)								
		75+	744	24‐h recall	7	(2)			18.7	(5.5)								
Canada	2004 37	70 + (M)	1520	24‐h recall	7.8	(5.5)	93	(117)	19.9									
		70 + (F)	2610	24‐h recall	6.3	(4.3)	82	(102)	21.8									
Finland	2007 66	65–74 (M)	229	48 h recall	7.7	(2.3)									44	(32)	9.3	(5.6)
		65–74 (F)	234	48 h recall	5.9	(1.7)									33	(26)	9.3	(5.1)
Iceland	2010–11 62	61–80 (M)	151	24‐h recall	8.7	(2.8)	80	(44)	15.4		35	(31)	6.5					
		61–80 (F)	167	24‐h recall	6.7	(2)	74	(32)	18.4		31	(24)	7.5					
Ireland	2008–10 63	65+	226	4 day record	7.4	(2.3)	84.1	(38)	17.9	(6.1)								
Italy	2005–6 41	65 + (M)	202	3 day record	9.6	(2.3)	81.6	(35)	13.3	(5)								
		65 + (F)	316	3 day record	7.7	(2)	78.6	(32)	16.2	(5.4)								
NZ	2008–9 58	71 + (M)	480	24‐h recall	8.1		105		21.8						45.9		9.5	
		71 + (F)	585	24‐h recall	6.1		84		23.0						33.5		9.2	
Norway	2010–11 64	60–70 (M)	217	24‐h recall	9.9	(2.9)					39	(32)	6.3	(4.4)				
		60–70 (F)	164	24‐h recall	7.4	(2.2)					30	(24)	6.5	(4.2)				
Sweden	2010–11 65	65–80	367	4 day diary	7.9	(2.2)	87.4		18.7		44.2	(25)	9.3	(4.3)	35.8	(19)	7.6	(3.3)
UK	2008–12 (Public Health England 2014)	65+	753	4 day diary	7.1	(2.05)	94.5	(41)	20.9	(6.4)	51.6	(33.4)	11.2	(5.6)				
US	2009–10 17	60–69 (M)	449	24‐h recall	9.2	(3.5)	108	(87)	19.6									
		70 + (M)	484	24‐h recall	8	(3.8)	104	(84)	21.8									
		60–69 (F)	453	24‐h recall	7.2	(3.2)	96	(70)	22.4									
		70 + (F)	513	24‐h recall	6.4	(3.3)	89	(50)	23.2									

M, males only; F, females only. Data are represented as the mean, unless otherwise indicated.

Where % energy was not given, this was calculated (using 0.016/0.017 MJg^−1^).

†Median values are given for the Netherlands.

‡Non‐milk extrinsic sugars (UK only) have been designated as added sugars.

a Data exclude under‐reporters (9.7%).

## Results

Tables 1, 2, 3, 4, 5 show the energy and sugars consumption in different age groups of the countries included in the present study. Of the 18 countries represented, the majority are in the continent of Europe (*n* = 13), with a good representation of North America (Canada and USA) and Australasia (Australia and New Zealand). There were only scarce data available from South America, with only limited information from Brazil, and no data available on countries in Africa or Asia. Data presented in the reports were collected at different time points, with the earliest data being collected in 1995 and the most recent data collected as recently as 2012.

The number of terms used to describe sugars has resulted in a literature on sugar intakes where data from study to study are not comparable. This limits any comparisons between countries and also impacts the ability to compare intakes with recommendations, risk factors or disease endpoints, and with the results of intervention studies. In the current review, all studies included estimates of energy intake, and the majority reported estimates of total sugars. Fewer reported intakes of added sugars or sucrose and no data were available on intakes of free sugars at the time the review was carried out.

Although most studies used the 24‐h recall method to collect dietary information, 3‐day food records were more common in surveys of infants and young children.

A small number of countries excluded under‐reporters from their analyses. These included those from Austria and France; however, the majority of studies did not indicate any adjustments for under‐reporting. Where these reporting data were available, the percentage of identified under‐reporters ranged from 2% to 25% in children and from 9% to 33% in adults. Some provision was made in the present review to minimise the effects of under‐reporting by reporting energy‐adjusted intakes, as well as absolute values for each country and sugars estimate. This is in keeping with findings from the literature that suggest including all subjects and using energy adjustment was preferable to excluding low‐energy reporters when reporting findings from self‐reported food intake data 13.

### Comparison across countries by age group

#### Infants and young children (<4 years)

For infants and young children, sugars consumption data are reported for 10 countries, including intakes of total sugars (eight countries), added sugars (five countries) and sucrose (Finland only) (Table 1). Total sugar intakes expressed as a percentage of total energy (%TE) ranged from 20% for 1‐year‐olds in Iceland to 38.4% for 4–6‐month‐olds in the UK. Intakes of added sugars (%TE) ranged from 1.9% for 9‐month‐olds in Iceland to 13.4% for 2–5‐year‐olds in the USA. Intake of sucrose (%TE) for Finland increased with age (1–3 years) from 5.8% for 1‐year‐olds to 13.4% for 3‐year‐olds.

#### Children (4–10 years)

For children aged 4–10 years, sugars consumption data are reported for 16 countries including intakes of total sugars (12 countries), added sugars (seven countries) and sucrose (five countries) (Table 2). Total sugar intakes expressed as a percentage of total energy (%TE) ranged from 17% in 3–10‐year‐olds in Italy to 34.8% for 4–6‐year‐old girls in the Netherlands. Intakes of added sugars (%TE) ranged from 9.0% for 5‐year‐olds in Iceland to 18% for 7–8‐year‐olds in the Netherlands. Intake of sucrose (%TE) ranged from 10.0% for 10–12‐year‐old boys in Austria to 17.0% for 7–8‐year‐old boys in The Netherlands.

#### Adolescents (12–18 years)

For older children aged 12–18 years, sugars consumption data are reported for 14 countries including intakes of total sugars (12 countries), added sugars (six countries) and sucrose (four countries) (Table 3). Total sugar intakes expressed as a percentage of total energy (%TE) ranged from 15.4% of total intake for 10–18‐year‐old boys in Italy to 29.6% for 15–17‐year‐old girls in Germany (food frequency questionnaire data). Intakes of added sugars (%TE) ranged from 12.4% for 13–17‐year‐olds in Ireland to 18.6% for 12–14‐year‐old girls in Norway. Intake of sucrose (%TE) ranged from 10% for 13–14‐year‐old boys in Austria to 16% for 9–13‐year‐old boys and girls in the Netherlands.

#### Adults (19–60 years)

For adults, sugars consumption data are reported for 18 countries including intakes of total sugars (13 countries), added sugars (nine countries) and sucrose (six countries) (Table 4). Total sugar intakes expressed as a percentage of total energy (%TE) ranged from 13.5% for 18–65‐year‐old men in Italy to 24.6% in 20–29‐year‐old women in the USA. Intakes of added sugars (%TE) ranged from 7.2% for >10‐year‐olds in Brazil and 7.3% for 18–70 years in Norway to 16.3% for 18–34‐year‐olds in the USA. Intake of sucrose (%TE) ranged from 7.7% for 18–80‐year‐olds in Sweden to 13.1% for 19–30‐year‐old women in New Zealand.

#### Older adults (>60 years)

For older adults, sugars consumption data are reported for 13 countries including intakes of total sugars (10 countries), added sugars (four countries) and sucrose (four countries) (Table 5). Total sugar intakes expressed as a percentage of total energy (%TE) ranged from 13.3% for >65‐year‐old men in Italy to 23.2% for >70‐year‐old women in the USA. Intakes of added sugars (%TE) ranged from 6.3% for 60–70‐year‐old men in Norway to 11.2% for >65‐year‐olds in the UK (NMES data). Intake of sucrose (%TE) ranged from 7.6% for 65–80‐year‐olds in Sweden to 9.5% for >71‐year‐old men in New Zealand.

**Figure 1:**
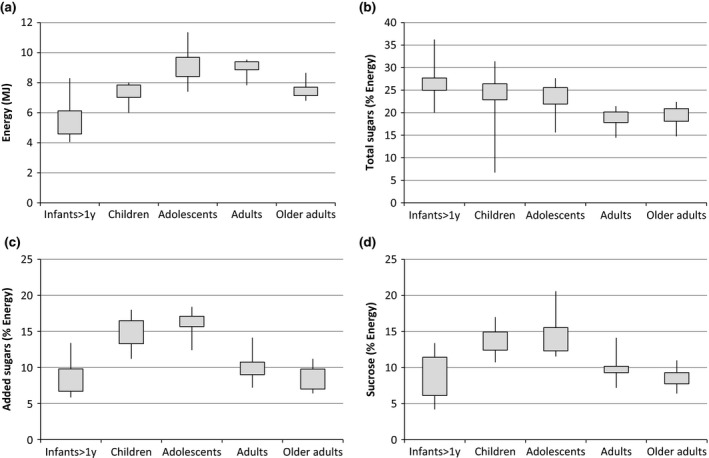
Crude boxplots of (a) energy intake (MJ), (b) total sugar intake (% Energy), (c) added sugar intake (% Energy) and (d) sucrose intake (% Energy). Data are collated from a number of different national dietary surveys across the world.

Figure 1 shows the crude range and distribution of energy and sugars consumption by age group. In general, total sugars as a percentage of energy are highest in the infant, with mean values ranging from 20.0% to 34.8%, and decrease over the lifespan to 14.5–20.5% in adults.

Intakes of added sugars were highest in adolescents, with mean values ranging from 12.4% to 18.6% of total energy. Mean intakes of added sugars were lower in infants and young children (<4 years), ranging from 1.9–13.4% and in older adults, ranging from 6.3% to 11.2%. A similar trend was observed for intake of sucrose; however, sucrose intake was only reported for four countries. For most countries surveyed, intakes of added sugars were greater than 10% for children and adolescents and less than 10% in infants and older adults.

## Discussion

The present review provides a resource of current dietary sugars intake in Europe, North America and Australasia based on data available from national dietary surveys and serves as a benchmark for on‐going surveillance. The disaggregation of the data by age‐band also allows us to draw some conclusions on sugars intake across the life‐span, from infants to older people.

Although the number of surveys undertaken in each age range varied, the data suggest that, in general, total sugars as a percentage of energy (%TE) are highest in infants and young children, with an average mean value of 28%, and decrease over the lifespan to 20% in adults and older adults. Data from the present review showed that, for young babies, relying totally on a milk‐based diet, 38% of energy came from total sugars 14. This decreases to 20–30% TE as weaning is introduced, with older infants obtaining their energy from a wider variety of sources. In terms of added sugars, the data available show that intakes of added sugars rise after age 1 year and added sugars intakes of greater than 10% were reported in pre‐school children in Australia, UK and the USA 15, 16, 17. For school‐aged children and adolescents, intakes of added sugars were consistently higher than that reported for younger children, with intakes of up to 19% TE reported. Intakes of added sugars then decreased across adulthood with intakes typically <10% TE in older adults. The large discrepancies that still exist with regard to both the definitions of sugars and the revised dietary guidance make it difficult to compare current intakes with recommendations; however, the data included in the present study clearly show that intakes of added sugars are highest in school‐aged children and adolescents. A recent review of trends in sugar intake worldwide has shown that, for adolescents, dietary intake of sugars has increased in some adolescent population groups (Dutch males) but has reduced in others (Denmark, USA, UK males and Dutch females) 18. Further research into the food groups and dietary patterns that are contributing to added sugar intakes in these population groups are warranted and will be useful in developing appropriate dietary guidelines.

The DGAC has previously recommended that national dietary surveys should report both total and added sugars and both classifications of sugars should be clearly defined allowing resulting analyses to determine whether it is meaningful to distinguish between the two when studying health outcomes 19. Almost all countries included in the present review reported intakes of total sugars and a considerable proportion reported an estimate of added sugars or non‐milk extrinsic sugars (UK). Although the international dietary guidance in relation to sugar consumption and oral health refers to a reduction in free sugars 3, none of the national surveys available at the time of the present study calculated intakes of free sugars using the WHO definition. Because total sugars can be analytically measured in foods, these values are included in most food composition databases and it is relatively straightforward for researchers to report intakes of total sugars based on food intake data. Estimates of total sugars, however, fall short of what is required to relate sugar intake to current dietary advice. This problem is essentially two‐fold. First, total sugars include sugars contained in milk (lactose) and fruit (fructose, glucose and sucrose) and most dietary recommendations encourage the consumption of both dairy and fruits as part of a healthy diet 20, 21. Second, because analytical methods cannot determine the difference between sugars naturally present in foods and those added at the processing stage 22, estimates of added or free sugars rely on information from food manufacturers that can be challenging to obtain and keep up to date. Indeed, the key reasons cited for the US Department of Agriculture decision to withdraw the added sugars component of the Survey Nutrient Database 23 were the frequent reformulation of products and the difficulty of obtaining proprietary information from manufacturers. Hence, obtaining reliable estimates of added and free sugar consumption remains a challenge for researchers and detailed food intake data at brand level is necessary for these purposes.

These specific considerations for sugars, combined with differences in national dietary survey methodologies and national clustering of age groups, all hinder the ability to compare consumption data across countries. To minimise these discrepancies, in Europe at least, the European Food Safety Authority is currently supporting a Pan‐European dietary survey methodology ‘EU MENU’ to harmonise food consumption data across Europe 24. EU Menu combines a standardised dietary assessment methodology with harmonised food composition data allowing participating countries to pool data, making comparisons on intakes of particular nutrients/foods more feasible in the future 24.

In the present study, the majority of the countries that are included collected dietary intake data using 24‐h recalls or dietary records. Both these dietary intake methods are suitable to estimate intakes of total sugars but have their limitations when assessing intakes of added or free sugars as discussed above. Both methods have their individual advantages and disadvantages, with dietary records being more accurate in terms of portion size and not as reliant on respondent memory, whereas 24‐h recalls are less prone to recording bias. Food frequency questionnaires (only used by one country in the present study) provide less accurate information in terms of absolute intakes but may provide valuable information in terms of habitual consumption of different foods/food groups. In a recent study reporting trends in sugar intake worldwide, a noted challenge was that the method of dietary analysis, which was employed in countries, frequently varied over time, and small changes in dietary intake of sugars could be a result of changes in the method of dietary analysis 18.

For all self‐reported food intake data, a generally accepted limitation is that of misreporting, where a proportion of individuals may alter their dietary pattern during the assessment or misreport (frequently under‐report) the foods and beverages they are consuming 25. In the surveys included in the present review, where reporting data were available, the percentage of identified under‐reporters (based on low‐energy reporting) ranged from 2% to 25% in children and from 9% to 33% in adults. For each country, in addition to reporting of absolute intakes, sugars were also expressed as a percentage of total energy. This approach can minimise the effect of under‐reporting; however, it cannot eliminate bias as a result of the selective under‐reporting of foods, nor does it provide corrected estimates of absolute nutrient intake 26.Under‐reporting can introduce bias if it is not uniform across food groups, age ranges or sex 27. For example, studies of children have shown that the proportion of under‐reporters markedly increased with the age of the child 28, 29. Furthermore, a secondary analysis of older adults in the UK National Diet and Nutrition Survey has shown that sex, body mass index and socio‐economic status affected the prevalence of under‐reporting 30. In this latter case, under‐reporting was found not to be uniform across different foods and food groups and suggested that alcohol and butter, as well as biscuits, cakes and pastries, were most likely to be under‐reported. Similarly, in a small study of female participants whose dietary intake was covertly measured over a 24‐h period, snack foods consumed between meals were under‐reported, as well as total carbohydrate and added sugars 31. A recent energy balance study has shown that misreporting self‐reported food intake data comprised two separate and synchronous phenomena. Subjects both under‐reported their food intake (reporting effect) and decreased their actual intake (observation effect) when they were aware that their intake was being monitored 32. Semi‐quantitative techniques gave larger discrepancies but, interestingly in that study, discrepancies were non‐macronutrient specific (for the reporting aspect). Further research is required to investigate whether specific food groups or nutrients such as sugars or sugar‐containing foods/beverages are more likely to be avoided as a result of observation effect.

Selection bias may also have an impact on the estimation of individual nutrient intakes, including that of sugars. Participation rates in the surveys included in the present study were generally in the region of 30–60%, indicating that, for some surveys at least, a significant proportion of the original sample frame declined to take part. Low response rates may indicate that the sample studied is not representative of the population because nonresponders may have different dietary patterns to those who chose to respond. For example, individuals who perceive themselves as having a less ‘healthy’ lifestyle may choose not to take part.

Notwithstanding the issues discussed above, which are commonly acknowledged with self‐reported dietary data, the present study provides both the most current and best estimates of sugar consumption for each population group in each country reported. Although the alternative means of estimating sugar intake (food supply data) plays an important role in tracking commodities, particularly from an economic perspective, it does not reflect actual intake, nor can it provide information on intake by sex, age range, region, or socio‐economic status, which is necessary for assessing the nutrient status of a population and devising nutrition policy. The authors acknowledge a marked gap in the present study with respect to the lack of available data from countries in the developing world, where it can be debated that the largest changes in sugar consumption may be occurring. Nations with rapid growth such as China and India have been widely reported to be consuming a more ‘Westernised diet’, and it would be of interest to see how this shift has affected sugar intake 33. Measuring nutrient intake in developing countries can be very challenging and sufficient funding can be hard to find, although successes are being achieved; for example, with the China Health and Nutrition Survey 34 and the Korean National Health and Nutrition Examination Survey 35 (although sugars intake was not reported in either survey).

To conclude, the present study provides a resource on current sugars consumption in different age ranges in various countries by collating data from nationally representative dietary surveys. Despite some differences in classification, the data included in this review suggest that, for many countries, intakes of added sugars in children and adolescents are higher than that of other population groups and further research into the dietary patterns contributing to these intakes is warranted. It would also be beneficial to policy guidance if future dietary surveys employed a uniform way of expressing added sugars that is feasible to measure and has public health significance.

**Conflict of interests, source of funding and authorship**

The authors declare that they have no conflicts of interest.

This review was funded by the World Sugar Research Organisation (WSRO) who had no role in the design, analysis or writing of this article. Katie Newens is an employee of Sugar Nutrition UK. Dr Janette Walton received a fee from WSRO for academic support of this review.

KN collated all the data and wrote the first draft of the paper. Both authors contributed to the plan of research, subsequent drafts of the manuscript, and approval of the final version submitted for publication.
